# Supplementary figures and images for: Research on image classification method based on improved multi-scale relational network
Source: PeerJ Comput Sci. 2021 Jul 21;7:e613. doi: 10.7717/peerj-cs.613 (PMC8323718; doi:10.7717/peerj-cs.613)

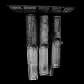

Supplement: Supplemental Information 1 — The multi-scale meta-relational network with the model-independent meta-learning algorithm. [file peerj-cs-07-613-s001.zip › RelationNetwork/miniimagenet/batch.jpg]

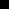

Supplement: Supplemental Information 1 — The multi-scale meta-relational network with the model-independent meta-learning algorithm. [file peerj-cs-07-613-s001.zip › RelationNetwork/miniimagenet/multi_feature24.jpg]

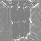

Supplement: Supplemental Information 1 — The multi-scale meta-relational network with the model-independent meta-learning algorithm. [file peerj-cs-07-613-s001.zip › RelationNetwork/miniimagenet/out1.jpg]

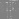

Supplement: Supplemental Information 1 — The multi-scale meta-relational network with the model-independent meta-learning algorithm. [file peerj-cs-07-613-s001.zip › RelationNetwork/miniimagenet/out2.jpg]

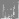

Supplement: Supplemental Information 1 — The multi-scale meta-relational network with the model-independent meta-learning algorithm. [file peerj-cs-07-613-s001.zip › RelationNetwork/miniimagenet/out3.jpg]

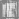

Supplement: Supplemental Information 1 — The multi-scale meta-relational network with the model-independent meta-learning algorithm. [file peerj-cs-07-613-s001.zip › RelationNetwork/miniimagenet/out4.jpg]
